# Association of a Novel Dietary Index Assessing Gut Microbiota Impact With Rheumatoid Arthritis: A Nationwide Population‐Based Study

**DOI:** 10.1002/fsn3.71932

**Published:** 2026-05-27

**Authors:** Zhi‐Ling Li, Yao‐Wei Zou, Pei‐Wen Jia, Ying Yang, Kui‐Min Yang, Hui‐Jing Li, Zhi‐Ming Ouyang

**Affiliations:** ^1^ Department of Rheumatology and Immunology The First People's Hospital of Zhaoqing Zhaoqing China; ^2^ Department of Rheumatology and Immunology, Sun Yat‐Sen Memorial Hospital Sun Yat‐sen University Guangzhou China

**Keywords:** diet, dietary index for gut microbiota, gut microbiota, NHANES, rheumatoid arthritis

## Abstract

Growing research implicates the diet‐microbiota‐joint axis plays a key role in joint health. The Dietary Index for Gut Microbiota (DI‐GM) is a novel metric evaluating dietary effects on microbial communities, yet its potential link to rheumatoid arthritis (RA) is unestablished. This study aimed to assess the relationship between DI‐GM scores and RA. We conducted a cross‐sectional analysis of 29,656 participants using data from the National Health and Nutrition Examination Survey (NHANES) 2007–2020. DI‐GM scores were derived from 24‐h dietary recalls, and RA status was defined by self‐reported physician diagnosis. Weighted logistic regression was used to evaluate the association between DI‐GM and RA prevalence. Among included participants (weighted mean age 44.01 years; 49.48% female), 7.16% reported RA. After comprehensive adjustment, a significant inverse correlation was observed: each unit increase in DI‐GM score correlated with a 5.7% decrease in the odds of RA prevalence (odds ratio [OR] = 0.943; 95% confidence interval [CI]: 0.898–0.991). Individuals scoring DI‐GM ≥ 7 exhibited a 27.2% lower odds of having RA compared to those scoring ≤ 4 (OR = 0.728, 95% CI: 0.576–0.920). Restricted cubic spline analysis indicated a linear dose–response relationship (*p* for nonlinear = 0.304). Furthermore, no significant subgroup interactions were detected (all *p* for interaction > 0.05). Our study found that higher DI‐GM scores linearly associate with diminished RA prevalence among US adults, highlighting a potential link between diet‐modulated gut microbiota and RA.

## Graphical abstract

This graphical abstract illustrates a cross‐sectional study of 29,656 U.S. adults from NHANES 2007–2020, examining the association between the Dietary Index for Gut Microbiota (DI‐GM) and rheumatoid arthritis (RA). Higher DI‐GM scores (reflecting beneficial foods for gut microbiota) were linearly associated with lower RA prevalence. Each 1‐point increase in DI‐GM corresponded to a 5.7% reduced odds of RA (OR = 0.943, 95% CI: 0.898–0.991). Participants with DI‐GM ≥ 7 showed a 27.2% lower RA risk relative to those with scores ≤ 4. These findings support a robust diet‐gut microbiota‐joint axis link, suggesting dietary modulation of gut microbiota may help lower RA risk.

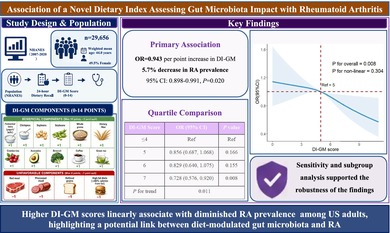

## 1 Background

Rheumatoid arthritis (RA), a chronic systemic autoimmune disorder, manifests through immune‐mediated invasion of synovial tissues, triggering progressive joint inflammation, cartilage degradation, and bone erosion that frequently culminate in functional disability (Di Matteo et al. 2023). Affecting an estimated 0.5%–1.0% of people globally, RA presents a substantial health challenge, with projections suggesting 31.7 million cases worldwide by 2050 (Black et al. 2023). Beyond joint damage, RA is strongly associated with serious comorbidities, including cardiovascular disease, pulmonary dysfunction, depression, and increased mortality risk (Figus et al. 2021). Emerging evidence highlights that individuals with RA face a 50%–60% higher mortality rate than the general population, intensifying the societal and healthcare burden (Logstrup et al. 2021). Understanding RA pathogenesis and modifiable risk factors is therefore essential for developing effective interventions.

The etiology of RA is multi‐factorial; genetic, immunological, hormonal, and environmental factors are important triggers (Di Matteo et al. 2023). Emerging research highlights a potential role for gut microbiota dysbiosis in RA pathogenesis, providing a promising avenue for RA treatment and prevention (Zaiss et al. 2021). Diet critically modulates gut microbiota composition and metabolic functions, with specific dietary patterns influencing host health via microbial activity (Hills Jr. et al. 2019). Based on a systematic review of 106 studies, Kase et al. developed the Dietary Index for Gut Microbiota (DI‐GM), incorporating 14 dietary components as influencing microbial profiles (Kase et al. 2024). Recent epidemiological applications of this index consistently show that higher DI‐GM scores, which reflect a diet that fosters microbial diversity and beneficial metabolites, are significantly associated with reduced risk of multiple chronic conditions, including diabetes (odds ratios [OR] = 0.93), fatty liver disease (OR = 0.94), depression (OR = 0.94), and stroke (OR = 0.93), underscoring its utility in capturing the systemic impact of diet‐microbiota interactions (Huang et al. 2025; Zheng et al. 2025; Zhang et al. 2024; Liu and Huang 2025). However, its potential association with RA risk remains unexplored.

This investigation utilized the DI‐GM to examine diet‐microbiota interactions concerning RA. Using the National Health and Nutrition Examination Survey (NHANES) data, it constitutes the first large‐scale assessment linking DI‐GM scores to RA, offering insights into the diet‐microbiota‐RA association.

## 2 Methods

### 2.1 Study Design and Population

The NHANES, administered by the CDC, employs a complex, multistage probability sampling design to collect nationally representative US health and nutrition data biennially (Prevention Centers for Disease Control and Centers for Disease Control and Prevention 2024). The National Center for Health Statistics Institutional Review Board approved the study, and all participants provided written informed consent.

**FIGURE 1:**
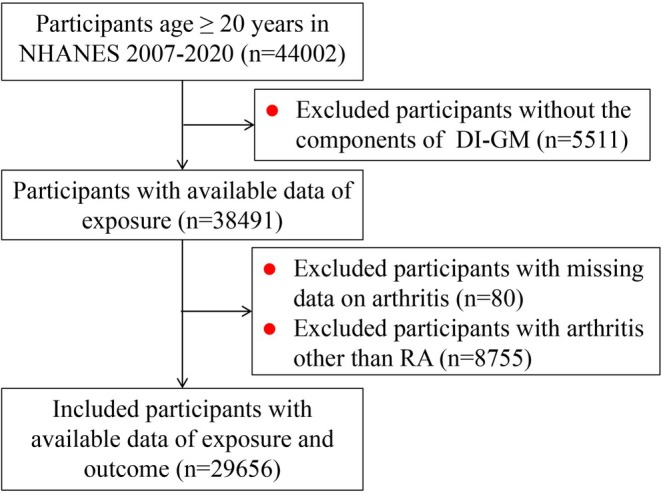
The flow chart of identifying eligible subjects. DI‐GM, dietary index for gut microbiota; NHANES, National Health and Nutrition Examination Survey; RA, rheumatoid arthritis.

From the analysis period of 2007–2020, of the initial 44,002 participants, 14,346 individuals were excluded due to missing DI‐GM (*n* = 5511) and arthritis data (*n* = 80), or non‐RA arthritis (*n* = 8755), leaving a primary analytic cohort of 29,656 subjects (Figure 1). Within this cohort, 5934 participants had missing values for certain covariates (e.g., marital status, family PIR, smoking status, alcohol intake, BMI or anti‐rheumatic therapy). While these individuals were excluded from the primary complete‐case analysis, they were retained for sensitivity analysis using multiple imputation to ensure the robustness of our findings.

### 2.2 Assessment of DI‐GM

Table S1 details DI‐GM components and scoring. This 14‐item index assesses:

- Beneficial Components (Max 10 points): Fermented dairy, chickpeas, soybeans, whole grains, dietary fiber, cranberries, avocados, broccoli, coffee, green tea. Intake ≥ sex‐specific median earns 1 point per item.
- Unfavorable Components (Max 4 points): Red meat, processed meat, refined grains, high‐fat diets (≥ 40% calories from fat). Intake ≥ sex‐specific median (or ≥ 40% fat) earns 0 points; lower intake earns 1 point per item.

Total scores range from 0 to 14. Participants were stratified by DI‐GM quartile: ≤ 4, 5, 6, ≥ 7.

### 2.3 Diagnosis of RA

RA diagnosis was based on self‐report survey responses. Participants answered two questions: (1) “Has a healthcare provider ever diagnosed you with arthritis?” and (2) “What type of arthritis was diagnosed?” Respondents who answered “no” to the first question were classified as non‐RA. Those who answered “yes” to the first and identified “RA” for the second were assigned to the RA group (Prevention Centers for Disease Control and Centers for Disease Control and Prevention 2024).

### 2.4 Assessment of Covariates

Demographic and lifestyle information was gathered through standardized surveys during home interviews. This included: age, gender, race/ethnicity (Mexican American, non‐Hispanic Black, non‐Hispanic White, non‐Hispanic Asian, other Hispanic, other races), education level (less than high school, high school graduate, college or higher), marital status (never married, married/living with partner, widowed/divorced/separated), family poverty income ratio (PIR), smoking status (never: < 100 lifetime cigarettes; current: ≥ 100 lifetime cigarettes; former: ≥ 100 lifetime cigarettes and quit), alcohol use (never: < 12 lifetime drinks; current: ≥ 12 drinks in past year; former: ≥ 12 lifetime drinks but no recent consumption). Weight and height were measured during physical exams, with body mass index (BMI, kg/m^2^) calculated as weight divided by height squared. Anti‐rheumatic medication use (yes/no) was recorded through standardized surveys.

### 2.5 Statistical Analysis

Analyses incorporated NHANES sample weights, stratification, and clustering for national representativeness. Categorical variables are presented as unweighted frequencies (weighted percentages), while continuous variables use weighted means with standard errors (SE). Group comparisons used weighted *t*‐tests for continuous data and weighted *χ* ^2^ tests with Rao‐Scott correction for categorical data.

Multivariate weighted logistic regression calculated OR and 95% confidence intervals (CI) for associations between DI‐GM scores (continuous/categorical) and RA. Nonlinear relationships were evaluated using restricted cubic splines (RCS) with knots at the 10th, 50th, and 90th percentiles. While four knots placed at the 5th, 35th, 65th, and 95th are common, we opted for the 10th, 50th, and 90th percentiles for our RCS model to achieve better stability in the spline estimation, especially given the discrete nature of the DI‐GM score (0–14). This placement allows for a more robust fit within the bulk of the data distribution while reducing potential noise from the extreme tails.

Sensitivity analyses included multiple imputation and propensity score matching (PSM). (1) Multiple imputation: Missing data (*n* = 5934) were imputed using chained equations, generating 5 datasets for repeated analyses. (2) PSM: 1:1 matching on RA status was performed to reduce confounding, followed by logistic regression on the matched sample. PSM were estimated using a logistic regression model in which RA status (yes/no) was regressed on a comprehensive set of baseline covariates, including age, sex, race/ethnicity, marital status, education level, PIR, smoking status, alcohol status, BMI, and anti‐rheumatic therapy. Matching was conducted using a caliper width of 0.1 times the standard deviation of the logit of the propensity score. Covariate balance after matching was evaluated using standardized mean differences (SMD); an SMD < 0.1 for all covariates was considered indicative of adequate balance (Table S2). All PSM analyses were implemented using the “MatchIt” package in R. Exploratory subgroup analyses were conducted across the following stratification: age (< 60 vs. ≥ 60 years), gender (male vs. female), race (non‐Hispanic White vs. others), education (less than college vs. college or above), marital status (married vs. others), family PIR (< 3.0 vs. ≥ 3.0), smoking (never/former vs. current), alcohol use (never/former vs. current), BMI (< 30 vs. ≥ 30 kg/m^2^), and anti‐rheumatic therapy (yes vs. no). It is important to note that these analyses were not adjusted for multiple comparisons and should therefore be considered exploratory and hypothesis‐generating. All analyses used R 4.2.3, with *p* < 0.05 indicating significance.

## 3 Results

### 3.1 Demographic and Clinical Characteristics of the Participants

From 44,002 eligible NHANES participants (2007–2020), exclusions for missing DI‐GM (*n* = 5511), missing arthritis data (*n* = 80), or non‐RA arthritis (*n* = 8755) yielded 29,656 participants in final analysis (Figure 1). Weighted characteristics are in Table 1. The mean age was 44.01 years; 49.48% were female. There were 4670 (9.65%) of Mexican American, 6820 (11.85%) non‐Hispanic Black, 10,899 (63.41%) non‐Hispanic White, 2819 (4.15%) non‐Hispanic Asian, and 4448 (10.94%) participants categorized as other races. Besides, 19.23% participants were current smokers and 72.71% were current drinkers. There were 0.82% participants with anti‐rheumatic therapy.

**TABLE 1:** Weighted demographic characteristics of participants according to DI‐GM score in NHANES.

Characteristics	Total (*n* = 29,656)	DI‐GM score				*p*
		≤ 4 (*n* = 12,767)	5 (*n* = 6889)	6 (*n* = 5145)	≥ 7 (*n* = 4855)	
Age, years, mean (SE)	44.01 (0.20)	42.59 (0.22)	43.64 (0.29)	44.82 (0.33)	46.78 (0.39)	< 0.001
Sex, *n* (%)						< 0.001
Male	15,109 (50.52)	6877 (54.58)	3469 (49.05)	2542 (49.84)	2221 (44.25)	
Female	14,547 (49.48)	5890 (45.42)	3420 (50.95)	2603 (50.16)	2634 (55.75)	
Race/ethnicity, *n* (%)						< 0.001
Mexican American	4670 (9.65)	2112 (11.10)	1210 (10.80)	766 (8.49)	582 (6.18)	
Non‐Hispanic Black	6820 (11.85)	3539 (15.20)	1542 (11.56)	1034 (9.96)	705 (6.84)	
Non‐Hispanic White	10,899 (63.41)	4270 (58.74)	2507 (62.74)	2048 (67.20)	2074 (70.57)	
Non‐Hispanic Asian	2819 (4.15)	884 (3.25)	616 (3.92)	561 (4.56)	758 (6.01)	
Other Hispanic	3197 (6.58)	1414 (7.40)	744 (6.63)	536 (5.98)	503 (5.34)	
Other Race	1251 (4.36)	548 (4.30)	270 (4.35)	200 (3.81)	233 (5.07)	
Educational level, *n* (%)						< 0.001
Less than high school	6647 (14.45)	3318 (17.72)	1643 (15.23)	992 (11.88)	694 (9.03)	
High school graduate	6753 (23.22)	3332 (28.09)	1605 (24.12)	1032 (19.65)	784 (15.16)	
College or above	16,226 (62.26)	6100 (54.19)	3635 (60.65)	3117 (68.47)	3374 (75.81)	
Marital status, *n* (%)						< 0.001
Never married	6379 (21.43)	3060 (23.92)	1474 (21.87)	1012 (20.01)	833 (16.90)	
Married/Living with Partner	17,668 (63.18)	7216 (60.00)	4130 (63.56)	3161 (64.80)	3161 (68.02)	
Widowed/Divorced/Separated	5594 (15.37)	2482 (16.07)	1283 (14.57)	969 (15.19)	860 (15.08)	
Family PIR, mean (SE)	3.01 (0.03)	2.74 (0.04)	2.98 (0.04)	3.16 (0.04)	3.49 (0.04)	< 0.001
Smoking status, *n* (%)						< 0.001
Never	17,420 (58.63)	7063 (55.76)	4173 (60.60)	3113 (59.34)	3071 (61.73)	
Former	6263 (22.12)	2624 (21.06)	1339 (20.29)	1131 (22.74)	1169 (26.15)	
Current	5959 (19.23)	3072 (23.19)	1376 (19.11)	897 (17.92)	614 (12.12)	
Alcohol intake, *n* (%)						0.01
Never	3645 (9.71)	1497 (10.84)	900 (11.58)	663 (10.32)	585 (9.31)	
Former	3058 (8.93)	1317 (9.70)	719 (10.33)	561 (10.19)	461 (8.82)	
Current	19,703 (72.71)	8470 (79.47)	4460 (78.09)	3423 (79.49)	3350 (81.87)	
BMI, kg/m^2^, mean (SE)	28.74 (0.08)	29.31 (0.11)	28.78 (0.11)	28.48 (0.13)	27.69 (0.15)	< 0.001
Anti‐rheumatic therapy, *n* (%)	255 (0.82)	97 (0.58)	62 (0.94)	51 (0.94)	45 (1.08)	< 0.001

Abbreviations: BMI, body mass index; DI‐GM, dietary index for gut microbiota; NHANES, National Health and Nutrition Examination Survey; PIR, poverty income ratio; SE, standard errors.

The DI‐GM score was right skewed distributed (median = 5.0, Interquartile range [IQR]: 4.0–6.0, Figure S1). We categorized the included sample into 4 groups based on the quartile of DI‐GM score (Table 1). Participants with a higher DI‐GM score were older, more often female, Non‐Hispanic White, current drinkers, with higher incomes and educational level and higher proportion of married or living with partner, using anti‐rheumatic drugs. However, there were lower proportion of current smokers and lower BMI in those with higher DI‐GM score.

### 3.2 Associations Between DI‐GM Score and RA

There were 2123 (7.16%) participants identified as RA. Notably, individuals with RA were older, more likely to be female and Non‐Hispanic Black, had a higher proportion of Widowed/Divorced/Separated, current smoking, former drinking, and using anti‐rheumatic drugs, but had lower incomes and educational attainment and BMI level (Table S2).

Table 2 shows significant inverse associations between DI‐GM score and RA prevalence in both unadjusted (OR = 0.957, 95% CI: 0.921–0.993, *p* = 0.021) and fully adjusted models (OR = 0.943, 95% CI: 0.898–0.991, *p* = 0.020), with each 1‐point increase linked to a 5.7% decrease in the odds of RA prevalence. Quartile analysis revealed similar trends: participants with DI‐GM score ≥ 7 had 27.2% lower RA odds compared to those with scores ≤ 4 (OR = 0.728, 95% CI: 0.576–0.920, *p* = 0.008). Fully adjusted models also showed that beneficial gut microbiota scores were associated with lower RA prevalence (OR = 0.926, 95% CI: 0.869–0.988, *p* = 0.020), while unfavorable gut microbiota scores showed no significant association after adjustments (Table 2).

**TABLE 2:** Weighted logistic analysis DI‐GM score with RA in participants in NHANES.

Characteristics	Crude model		Adjusted model	
	OR (95% CI)	*p*	OR (95% CI)	*p*
DI‐GM score	0.957 (0.921, 0.993)	0.021	0.943 (0.898, 0.991)	0.020
Stratified by DI‐GM score				
≤ 4	Ref		Ref	
5	0.927 (0.788, 1.092)	0.362	0.856 (0.687, 1.068)	0.166
6	0.897 (0.729, 1.104)	0.303	0.829 (0.640, 1.075)	0.155
≥ 7	0.812 (0.669, 0.986)	0.036	0.728 (0.576, 0.920)	0.008
*p* for trend		0.037		0.011
Beneficial to gut microbiota score	0.912 (0.870, 0.955)	< 0.001	0.926 (0.869, 0.988)	0.020
Unfavorable to gut microbiota score	1.054 (0.990, 1.122)	0.097	0.985 (0.912, 1.063)	0.693

*Note:* The crude model was not adjusted for any covariates, while the adjusted model was adjusted for age, sex, race/ethnicity, education level, marital status, family PIR, smoking status, drinking status, BMI and anti‐rheumatic therapy.

Abbreviations: BMI, body mass index; CI, confidence intervals; DI‐GM, dietary index for gut microbiota; NHANES, National Health and Nutrition Examination Survey; OR, odds ratios; PIR, poverty income ratio; RA, rheumatoid arthritis.

**FIGURE 2:**
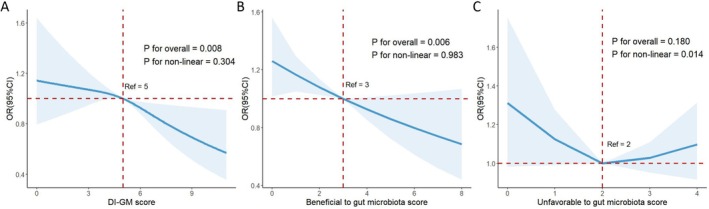
Nonlinear dose–response analysis on the association of DI‐GM score with RA in participants in NHANES. BMI, body mass index; CI, confidence intervals; DI‐GM, dietary index for gut microbiota; NHANES, National Health and Nutrition Examination Survey; OR, odds ratios; PIR, poverty income ratio; RA, rheumatoid arthritis. * Adjusted for age, sex, race/ethnicity, education level, marital status, family PIR, smoking status, drinking status, BMI, and anti‐rheumatic therapy.

RCS analysis confirmed linear relationships between DI‐GM score and RA prevalence (*p*_nonlinear_ = 0.304), as well as between beneficial to gut microbiota scores and RA (*p*_nonlinear_ = 0.983, Figure 2).

### 3.3 Sensitivity Analyses and Subgroup Analyses

**FIGURE 3:**
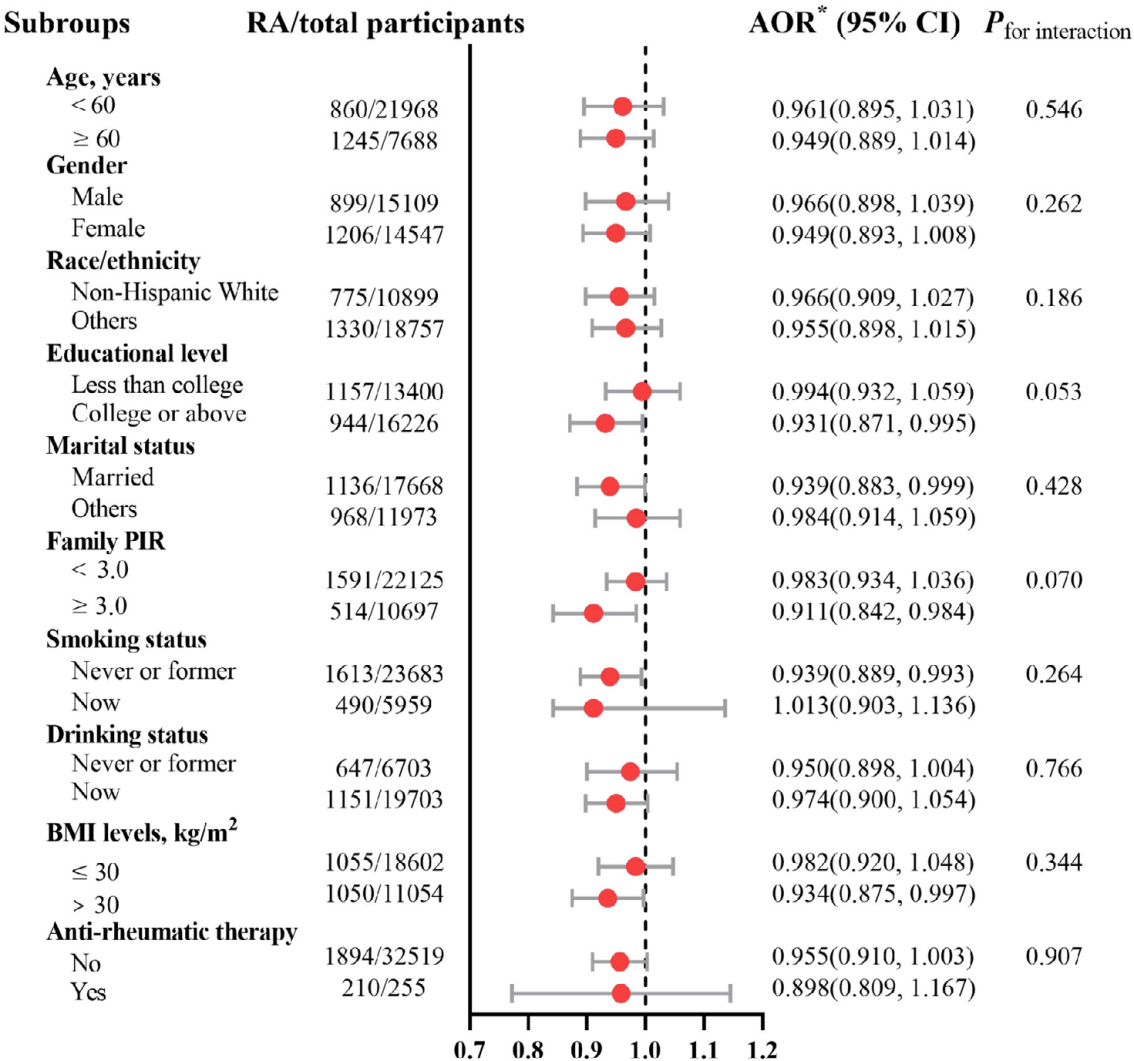
Associations of DI‐GM score with RA in various subgroups in participants in NHANES. AOR, adjusted odds ratios; BMI, body mass index; CI, confidence intervals; DI‐GM, dietary index for gut microbiota; NHANES, National Health and Nutrition Examination Survey; PIR, poverty income ratio; RA, rheumatoid arthritis. *Adjusted for age, sex, race/ethnicity, education level, marital status, family PIR, smoking status, drinking status, BMI, and anti‐rheumatic therapy.

In sensitivity analyses, results from multiple imputation showed that the negative association between DI‐GM and RA remained significant in the adjusted model (OR = 0.950, 95% CI: 0.910–0.991, *p* = 0.018). PSM analysis (OR = 0.942, 95% CI: 0.890–0.997, *p* = 0.040, Table S3) confirmed this association, supporting the robustness of the primary findings. Furthermore, we performed exploratory subgroup analyses across various strata to evaluate the consistency of the association between DI‐GM scores and RA (Figure 3). The results showed that there was no significant effect modification (*p* for interaction > 0.05).

## 4 Discussion

This nationwide study firstly establishes an inverse relationship between DI‐GM scores and RA prevalence, demonstrating a 5.7% decrease in the odds of RA prevalence per 1‐point DI‐GM increase. These results highlight the complex interplay between diet, gut microbes, and RA development, contributing to a better understanding of the interplay between diet, gut microbiota, and RA.

RA is a systemic autoimmune disorder involving genetic susceptibility, environmental factors, and immune dysfunction (Di Matteo et al. 2023). Genome‐wide association studies have identified over 100 RA risk genes, with HLA‐DRB1 alleles playing a central role in disease development by affecting antigen presentation. However, genetic factors account for only ~30% of RA risk, emphasizing the contribution of environmental factors (e.g., smoking, diet) via genetic modifications and microbiota remodeling (Okada et al. 2014). Recent research shows gut microbial imbalance contributes to RA pathogenesis, and diet significantly influences gut microbiota composition (Zaiss et al. 2021; Hills Jr. et al. 2019). In this study, we employed a novel tool (DI‐GM score) to quantify diet‐microbiota interactions (Kase et al. 2024), and found a robust inverse relationship between DI‐GM scores and RA prevalence. Our findings suggest an association between dietary patterns that favorably modulate gut microbiota and lower prevalence of RA, supporting the diet‐microbiota‐RA axis as a potential area for further investigation.

It has been suggested that interventions targeting the gut microbiota, such as dietary changes, might reduce RA risk by restoring microbial balance. For example, long‐term adherence to healthy diet patterns (assessed by the Healthy Eating Index (HEI)‐2010/2015, or Mediterranean diet) was significantly associated with reduced RA risk (Hu et al. 2017; Kanika et al. 2024; Nguyen et al. 2021). Specifically, long‐chain omega‐3 polyunsaturated fatty acids found in oily fish (Di Giuseppe et al. 2014), moderate alcohol use (Hedenstierna et al. 2021), vegetables (Philippou and Nikiphorou 2018), and olive oil (Philippou and Nikiphorou 2018) have been associated with reduced RA risk. Conversely, high‐sugar/fat diet and red meat have been associated with higher RA risk (Philippou and Nikiphorou 2018; Jiang et al. 2022). Our findings support these views, showing higher DI‐GM scores (reflecting beneficial gut microbiota components like fiber and phytochemicals) linked to lower RA prevalence. Possible mechanisms involve regulation of inflammatory pathways, gut microbial composition, or immune responses, and gut microbiota likely mediates the diet‐RA relationship (Sparks and Costenbader 2018).

Interestingly, while the total DI‐GM score and the “beneficial to gut microbiota” subscore showed significant inverse associations with RA, the “unfavorable to gut microbiota” subscore, comprising red meat, processed meat, refined grains, and high‐fat intake, did not maintain statistical significance after comprehensive adjustment (OR = 0.985, *p* = 0.693). This null result warrants careful consideration, as existing literature often emphasizes the pro‐inflammatory potential of such dietary components in autoimmune pathology (Philippou and Nikiphorou 2018; Jiang et al. 2022). One plausible explanation is that the protective signal within the DI‐GM is predominantly driven by the active presence of microbiota‐supporting foods, such as fiber and phytochemicals, which may exert a more potent immunomodulatory effect than the mere absence of unfavorable foods. On the other hand, a previous study showed that the “unfavorable” foods may require a specific microbial environment, or a lack of protective fiber, to exert their pro‐inflammatory effects (Jiang et al. 2022). This suggests that for RA prevention, increasing the intake of beneficial, microbiota‐friendly foods may be as critical, if not more so, than simply restricting pro‐inflammatory dietary elements.

Gut microbiota acts as environmental sensors, mediating systemic immune imbalance through the “gut‐joint axis” (Zaiss et al. 2021). Studies have revealed that RA patients often experience dysbiosis of gut microbiota, characterized by reduced diversity, overgrowth of pro‐inflammatory species (e.g., *Prevotella copri* and *Collinsella*), and depletion of beneficial taxa (e.g., *Faecalibacterium*), which can lead to loss of immune tolerance, worsening joint inflammation (Horta‐Baas et al. 2017). For instance, *Collinsella* enhances gut permeability and IL‐17A production, while *Prevotella copri* promotes Th17 cell polarization, amplifying synovial inflammation (Chen et al. 2016; Pianta et al. 2017; Nii et al. 2023). Molecular mimicry between microbial antigens (e.g., *Porphyromonas gingivalis* enzymes) and citrullinated host proteins further fuels auto‐antibody generation, bridging mucosal immunity to joint pathology (Wegner et al. 2010; Gomez‐Banuelos et al. 2022). Concurrently, gut microbiota‐derived metabolites modulate disease progression. Short‐chain fatty acids (SCFAs) like *butyrate* reduce inflammation by enhancing regulatory T cells, yet RA patients exhibit diminished SCFA levels, correlating with disease severity (Luu and Visekruna 2019; Cao et al. 2024). Conversely, altered tryptophan metabolism and bile acid modifications by microbes like *Bacteroides fragilis* may accelerate osteoclast activity and fibroblast growth (Lin et al. 2023; Ouyang et al. 2025). These findings highlight gut microbiota as a potential therapeutic target for RA. As synthesized in a recent comprehensive review, modulating the gut microbiota through targeted dietary strategies and therapeutic interventions represents a promising “novel treatment avenue” for RA by restoring the gut‐joint axis and mitigating systemic inflammation (Chasov et al. 2024). However, it is important to note that since gut microbiota composition was not directly measured in the NHANES cohort, the above mechanistic pathways remain speculative and are intended to be hypothesis‐generating rather than explanatory of our specific findings. Nonetheless, these specific microbial interactions highlight the importance of dietary patterns in modulating gut microbiome composition and function. Notably, the DI‐GM index captures key dietary components that are plausibly linked to these microbial mechanisms, for instance, high fiber and whole grain intake promotes the expansion of butyrate‐producing taxa that strengthen the intestinal barrier, while fermented dairy and phytochemical‐rich foods like green tea may enhance microbial diversity and suppress pro‐inflammatory species like *Prevotella copri* . By capturing these synergistic interactions, the DI‐GM serves as a functional proxy for a “gut‐healthy” dietary pattern that mitigates the systemic immune activation typical of RA.

Our findings have important clinical implications. This study establishes the first adjusted association between DI‐GM scores and RA prevalence, suggesting that dietary patterns reflected by higher DI‐GM scores are associated with lower RA prevalence, warranting further investigation in longitudinal studies. However, there were still several limitations. First, because the analysis is cross‐sectional, we cannot determine whether dietary patterns preceded RA diagnosis. It is possible that individuals changed their diets after being diagnosed, which could lead to higher DI‐GM scores and bias the observed association toward the null or even produce a misleading inverse relationship. Second, RA status was based on self‐report without clinical confirmation. While this approach is common in large surveys like NHANES, it may result in misclassification, which likely attenuates the true association. In addition, we observed a high concentration of RA cases within the anti‐rheumatic therapy subgroup (unweighted *n* = 210/255, 82.4%), which represents an inherent ascertainment bias as these medications are typically prescribed following a clinical diagnosis. Nonetheless, the consistent association observed across all subgroups supports the robustness of the DI‐GM as a credible exposure variable. Third, diet was assessed using two 24‐h recalls. Although we used the National Cancer Institute method to estimate usual intake, random day‐to‐day variation and systematic reporting errors may still introduce non‐differential measurement error, further weakening observed associations. Forth, though we adjusted for multiple socio‐demographic and lifestyle factors, certain potential confounders known to influence both the gut microbiota and RA (such as physical activity, the use of antibiotics or proton pump inhibitors, and oral health status) were not included in our primary models, and their potential to confound the observed dietary associations should be considered in future prospective study. Taken together, these limitations mean our findings reflect only an association with prevalent, self‐reported RA and do not support causal or preventive interpretations. Prospective studies with clinically verified RA and repeated dietary measures are needed to clarify these relationships.

## 5 Conclusions

In conclusion, this study found that higher DI‐GM score is associated with lower RA prevalence among US adults. Our findings suggest that dietary patterns as assessed by the DI‐GM may play a protective role in the systemic inflammation underlying RA, likely through their influence on the gut‐joint axis. However, due to the cross‐sectional design, lack of clinical RA confirmation, and potential for reverse causation, these findings should be interpreted as hypothesis‐generating rather than indicative of a causal or preventive relationship. Future prospective studies integrating high‐resolution dietary tracking or Mendelian randomization are required to establish causality for microbiota‐targeted dietary interventions in RA.

## Author Contributions

**Yao‐Wei Zou:** conceptualization, methodology, software, data curation, investigation, funding acquisition, writing – original draft. **Pei‐Wen Jia:** conceptualization, methodology, software, data curation. **Ying Yang:** conceptualization, methodology, software, data curation. **Zhi‐Ming Ouyang:** conceptualization, methodology, software, data curation, supervision, writing – review and editing, investigation. **Zhi‐Ling Li:** conceptualization, methodology, investigation, writing – original draft, software, data curation. **Kui‐Min Yang:** conceptualization, methodology, software, data curation. **Hui‐Jing Li:** conceptualization, methodology, software, data curation, writing – review and editing, investigation, supervision.

## Funding

National Natural Science Foundation of China (82402094); Traditional Chinese Medicine Bureau of Guangdong Province Research Project (20251061); Sun Yat‐sen Pilot Scientific Research Fund (YXQH202604).

## Ethics Statement

Approved by NCHS IRB; conducted per Declaration of Helsinki.

## Consent

Written informed consent obtained.

## Supporting information


**Figure S1:** The distribution of DI‐GM score in NHANES;
**Table S1:** Components and scoring criteria of DI‐GM in NHANES;
**Table S2:** Weighted demographic characteristics of participants according to RA status in NHANES;
**Table S3:** Association between DI‐GM with RA of the NHANES participants after multiple imputation of missing data or propensity score matching.

## Data Availability

The data that support the findings of this study are available on request from the corresponding author. The data are not publicly available due to privacy or ethical restrictions.
