# Supplementary material for: Association of a Novel Dietary Index Assessing Gut Microbiota Impact With Rheumatoid Arthritis: A Nationwide Population‐Based Study
Source: Food Sci Nutr. 2026 May 27;14(6):e71932. doi: 10.1002/fsn3.71932 (PMC13240211; doi:10.1002/fsn3.71932)
Supplement: Supplementary file 1 — Figure S1: The distribution of DI‐GM score in NHANES; Table S1: Components and scoring criteria of DI‐GM in NHANES; Table S2: Weighted demographic characteristics of participants according to RA status in NHANES; Table S3: Association between DI‐GM with RA of the NHANES participants after multiple imputation of missing data or propensity score matching. [file FSN3-14-e71932-s001.docx]

**Supplementary Materials**

**Supplementary Table S1 Components and scoring criteria of DI-GM in NHANES**

| Components of DI-GM | Food items included in NHANES | Scoring criteria | Sex-specific median cutoffs | |
| --- | --- | --- | --- | --- |
|  |  |  | Male | Female |
| Beneficial to gut microbiota | Avocados (g) | Score 1 - Consumption ≥ sex-specific median  Score 0 - Otherwise | 9.95 | 12.51 |
|  | Broccoli (g) |  | 63.78 | 58.77 |
|  | Chickpeas (g) |  | 5.10 | 4.87 |
|  | Coffee (g) |  | 385.51 | 328.19 |
|  | Cranberries (g) |  | 49.74 | 37.85 |
|  | Fermented dairy (including yogurt, cheese, kefir, sour cream, buttermilk) (g) |  | 403.12 | 329.76 |
|  | Fiber (g) |  | 18.75 | 15.46 |
|  | Soybean (including Soy milk, Tofu) (g) |  | 80.01 | 76.10 |
|  | Whole grains (oz) |  | 0.88 | 0.77 |
| Unfavorable to gut microbiota | Refined grains (oz) | Score 0 - Consumption ≥ sex-specific median  Score 1 - Otherwise | 6.90 | 5.02 |
|  | Processed meat (oz) |  | 1.28 | 0.72 |
|  | Red meat (oz) |  | 3.16 | 1.73 |
|  | High-fat diet (% energy) | Score 0 - Consumption ≥ 40%  Score 1 - Otherwise | 38.42 | 23.14 |

Abbreviations: NHANES, National Health and Nutrition Examination Survey; DI-GM, dietary index for gut microbiota; oz, ounce; g, gram.

**Supplementary Table S2 Weighted demographic characteristics of participants according to RA status in NHANES**

| Characteristics | Before propensity score matching | | |  | After propensity score matching | | | |
| --- | --- | --- | --- | --- | --- | --- | --- | --- |
|  | Without RA (n=27533) | RA  (n=2113) | *P* value |  | Without RA (n=1913) | RA  (n=1913) | *P* value | SMD |
| Age, years, mean (SE) | 43.28(0.20) | 57.54(0.40) | <0.001 |  | 58.36(0.59) | 57.37(0.44) | 0.92 | 0.02 |
| Sex, n (%) |  |  | <0.001 |  |  |  | 0.91 | 0.003 |
| Male | 14201(50.97) | 908(42.17) |  |  | 853(40.55) | 856(44.58) |  |  |
| Female | 13332(49.03) | 1215(57.83) |  |  | 1060(59.45) | 1057(55.42) |  |  |
| Race/ethnicity, n (%) |  |  | <0.001 |  |  |  | 0.56 | 0.04 |
| Mexican American | 4397(9.76) | 273(7.59) |  |  | 224(6.28) | 252(7.92) |  |  |
| Non-Hispanic Black | 6129(11.55) | 691(17.33) |  |  | 610(17.51) | 618(17.33) |  |  |
| Non-Hispanic White | 10119(63.43) | 780(63.02) |  |  | 749(65.10) | 697(62.08) |  |  |
| Non-Hispanic Asian | 2740(4.27) | 79(1.99) |  |  | 54(1.43) | 68(2.00) |  |  |
| Other Hispanic | 2971(6.62) | 226(5.84) |  |  | 207(5.64) | 211(6.15) |  |  |
| Other Race | 1177(4.37) | 74(4.23) |  |  | 69(4.04) | 67(4.52) |  |  |
| Marital status, n (%) |  |  | <0.001 |  |  |  | 0.73 | 0.01 |
| Never married | 6169(22.12) | 210( 8.72) |  |  | 183(8.30) | 189(8.64) |  |  |
| Married/Living with Partner | 16520(63.30) | 1148(61.14) |  |  | 1035(59.99) | 1052(61.52) |  |  |
| Widowed/Divorced/Separated | 4830(14.57) | 764(30.14) |  |  | 692(31.61) | 671(29.82) |  |  |
| Educational level, n (%) |  |  | <0.001 |  |  |  | 0.73 | 0.01 |
| Less than high school | 6001(14.06) | 646(21.79) |  |  | 574(20.58) | 607(23.00) |  |  |
| High school graduate | 6238(23.02) | 515(27.13) |  |  | 484(26.42) | 464(27.31) |  |  |
| College or above | 15268(62.92) | 958(51.08) |  |  | 849(52.80) | 838(49.58) |  |  |
| PIR, mean (SE) | 3.04(0.03) | 2.54(0.06) | <0.001 |  | 2.61(0.06) | 2.48(0.07) | 0.66 | 0.02 |
| Smoking status, n (%) |  |  | <0.001 |  |  |  | 0.97 | 0.008 |
| Never | 16439(59.39) | 981(44.99) |  |  | 892(47.35) | 884(45.19) |  |  |
| Former | 5615(21.62) | 648(31.39) |  |  | 564(29.31) | 571(30.45) |  |  |
| Current | 5467(18.99) | 492(23.62) |  |  | 454(23.25) | 456(24.30) |  |  |
| Alcohol intake, n (%) |  |  | <0.001 |  |  |  | 0.60 | 0.05 |
| Never | 3359(10.56) | 286(11.83) |  |  | 255(12.20) | 255(10.69) |  |  |
| Former | 2691( 9.28) | 367(18.88) |  |  | 356(16.50) | 330(16.96) |  |  |
| Current | 18540(80.16) | 1163(69.28) |  |  | 1061(61.70) | 1053(62.80) |  |  |
| BMI, kg/m^2^, mean (SE) | 28.62(0.08) | 30.80(0.24) | <0.001 |  | 30.43(0.30) | 30.66(0.24) | 0.63 | 0.02 |
| Anti-rheumatic therapy, n (%) | 27( 0.14) | 228(13.47) | <0.001 |  | 27(2.53) | 32(2.58) | 0.84 | 0.02 |

Abbreviations: NHANES, National Health and Nutrition Examination Survey; RA, rheumatoid arthritis; PIR, poverty income ratio; BMI, body mass index; SE, standard errors.

**Supplementary Table S3 Association between DI-GM with RA of the NHANES participants after multiple imputation of missing data or propensity score matching**

| Characteristics | Multiple imputations of missing data | |  | Propensity score matching | | |
| --- | --- | --- | --- | --- | --- | --- |
|  | OR (95% CI)* | *P* value |  | OR (95% CI)* | *P* value |  |
| DI-GM score | 0.950(0.910, 0.991) | 0.018 |  | 0.942(0.890, 0.997) | 0.040 |  |
| Stratified by DI-GM score |  |  |  |  |  |  |
| ≤ 4 | Ref |  |  | Ref |  |  |
| 5 | 0.889(0.735, 1.076) | 0.225 |  | 0.803(0.627, 1.029) | 0.082 |  |
| 6 | 0.870(0.689, 1.098) | 0.237 |  | 0.859(0.651, 1.133) | 0.277 |  |
| ≥ 7 | 0.768(0.622, 0.947) | 0.014 |  | 0.749(0.574, 0.977) | 0.033 |  |
| *P* for trend |  | 0.015 |  |  | 0.029 |  |
| Beneficial to gut microbiota score | 0.938(0.888, 0.991) | 0.023 |  | 0.944(0.883, 1.009) | 0.091 |  |
| Unfavorable to gut microbiota score | 0.982(0.918, 1.052) | 0.604 |  | 0.961(0.873, 1.057) | 0.409 |  |

Abbreviations: NHANES, National Health and Nutrition Examination Survey; DI-GM, dietary index for gut microbiota; RA, rheumatoid arthritis; PIR, poverty income ratio; BMI, body mass index; OR, odds ratios; CI, confidence intervals.

* Adjusted for age, sex, race/ethnicity, education level, marital status, family PIR, smoking status, drinking status, BMI and anti-rheumatic therapy.


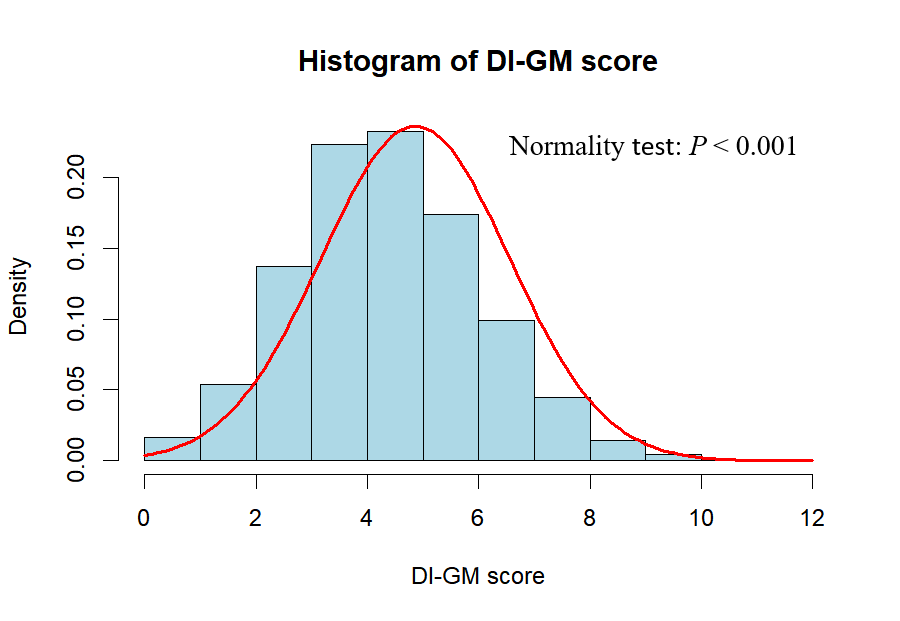


**Supplementary Figure S1 The distribution of DI-GM score in NHANES**

Abbreviations: NHANES, National Health and Nutrition Examination Survey; DI-GM, dietary index for gut microbiota.
